# Sensitivity Analysis of *C. auris, S. cerevisiae*, and *C. cladosporioides* by Irradiation with Far-UVC, UVC, and UVB

**DOI:** 10.20411/pai.v9i2.723

**Published:** 2024-08-21

**Authors:** Anna-Maria Gierke, Martin Hessling

**Affiliations:** 1 Institute of Medical Engineering and Mechatronics, Ulm University of Applied Sciences, Ulm, Germany

**Keywords:** 222 nm, 254 nm, 302 nm, disinfection, photoinactivation, surrogate

## Abstract

**Background:**

The World Health Organization has published a list of pathogenic fungi with prior-itizing groups and calls for research and development of antifungal measures, with *Candida auris* belonging to the group with high priority.

**Methods:**

The photosensitivity towards short wavelength ultraviolet irradiation (Far-UVC, UVC, and UVB) was investigated and compared to other yeasts (*Saccharomyces cerevisiae*) and a mold (*Cladosporium cladosporioides*). The observed 1-log reduction doses were compared to literature values of other representatives of the genus *Candida*, but also with *S. cerevisiae, Aspergillus niger,* and *A. fumigatus*.

**Results:**

For the determined 1-log reduction doses, an increase with higher wavelengths was observed. A 1-log reduction dose of 4.3 mJ/cm^2^ was determined for *C. auris* when irradiated at 222 nm, a dose of 6.1 mJ/cm^2^ at 254 nm and a 1-log reduction dose of 51.3 mJ/cm^2^ was required when irradiated with UVB.

**Conclusions:**

It was observed that *S. cerevisiae* is a possible surrogate for *C. auris* for irradiation with Far-UVC and UVB due to close 1-log reduction doses. No surrogate suitability was verified for *C. cladosporioides* in relation to *A. niger* and *A. fumigatus* for irradiation with a wavelength of 254 nm and for *A. niger* at 222 nm.

## INTRODUCTION

In 2022, the World Health Organization (WHO) published a document listing important fungal pathogens in groups of different importance. *Candida auris* was classified with *C. albicans, Aspergillus fumigatus* and *Cryptococcus neoformans* as the critical priority group [1]. Due to the increasing development of antifungal drug resistance, treatment of infection has become more difficult [2–5]. *Candida* spp. are known to cause invasive fungal infections (IFIs) [2, 6, 7]. In addition to the very wide-spread representative *Candida albicans*, the number of infected persons and deaths worldwide due to *C. auris* have been increasing over the last few years. As was first described in Japan, *C. auris* has been discovered in hospitals and healthcare facilities in more countries [2, 8–10]. It is a lethal threat, especially to immunocompromised patients [11, 12].

In addition to antimycotics, there are also studies on chemical disinfectants that are intended to reduce the spread of nosocomial infections [13–15]. Another possibility for the reduction of *C. auris* and other pathogenic fungi is irradiation at different wavelengths. For the selected spectral ranges, damage to humans by application of this radiation to different body areas is described by Ramasamy et al [16]. Due to short wavelength UV irradiation, cyclobutane pyrimidine dimers (CPD) and 6-4 photoproducts in DNA (deoxyribonucleic acid) are formed, which can result in mutagenic effects or even cell death [16–21].

In the case of UVC, however, a further distinction should be made on the basis of recent studies on the effect of the so-called Far-UVC (200 - 230 nm) [22, 23]. In contrast to an irradiation at 254 nm, which is hazardous to humans, an application of Far-UVC radiation on human skin and eyes appears less harmful [16, 17, 24, 25] and might even allow radiation disinfection in the presence of humans.

In this study, the 1-log reduction doses for *C. auris* are determined and compared with the D90 values of other representatives of the genus Candida, another yeast (*S. cerevisiae*) and molds with pigmented spores (*C. cladosporioides, A. niger* and *A. fumigatus*). The experiments are carried out in transparent liquid suspensions to obtain information about photoinactivation based only on fungal properties. Further 1-log reduction doses are taken from other studies with similar experimental setups. The aim is to make statements about possible surrogates for irradiation experiments for *C. auris* and *C. albicans* or also about similarities and differences between the examined fungi. Furthermore, it should be investigated whether there is a possible connection between pathogenicity and radiation sensitivity.

When irradiating the pigmented spores of the mold, 1-log reduction doses for *C. cladosporioides* that are many times higher are expected and compared to *A. niger*, whose genus is also classified as potentially pathogenic [1, 26, 27] and *A. fumigatus,* which is listed by WHO in the critical priority group [1]. This is intended to consider whether *C. cladosporioides* could be a possible surrogate for *A. niger* or *A. fumigatus. C. cladosporioides* is chosen for experiments due to its very wide distribution and pigmented spores protecting against radiation damage.

## METHODS

The following fungal strains were investigated in the irradiation experiments: *C. auris* (DSM 21092, clade II, ATCC MYA-5001, strain designation B11220), *S. cerevisiae* (DSM 70449) and *C. cladosporioides* (DSM 19653). Since the present laboratory has a biosafety level of 1 and work with pathogenic representatives of yeasts and molds is only permitted in laboratories with higher safety standards, these fungi were selected.

For the 2 yeasts *C. auris* and *S. cerevisiae*, modified YEPG (Yeast Extract Peptone Glucose medium - 200 ml glucose (250 g/l), 20 g peptone from casein, 10 g yeast extract per 1,000 ml; pH value of 6.5) was used for the liquid cultures and potato dextrose agar (M129 – 20 g dextrose, 4 g potato extract, [15 g agar] per 1,000 ml; pH 5.6) for the agar plates. For the mold *C. cladosporioides*, M90 (30 g malt extract, 3 g soya peptone, [15 g agar] per 1,000 ml; pH 5.6) was chosen for agar plates and liquid cultures. Both yeasts were cultured at 30 °C until mid-exponential phase resulting in a population density of around 3 x 10^9^ CFU/ml (colony forming units per ml). After reaching the growth phase, 3 ml of the culture was removed and centrifuged at 7,000 rpm for 5 minutes. The resulting supernatant was discarded and the pellet resuspended in phosphate buffered saline (PBS). The washing procedure was repeated twice. The sample was then diluted to a population density of 5 x 10^6^ to 1 x 10^7^ colony forming units (CFU) per mL. The sample transmission for the irradiation wavelength was measured in a 10 mm quartz cuvette using a spectrophotometer (SPE-CORD 250 PLUS double beam spectrophotometer, Analytik Jena GmbH+Co. KG) to ensure a transmission of 70% to 80% for a sample thickness of 3 mm, the sample height during irradiation. In the case of the mold, only the spores were investigated. For this purpose, spores were previously spread on an agar plate and allowed to grow at 24 °C for 4 days. A solution of 0.9% NaCl and 0.01% TWEEN 80 was then added to the overgrown plate to brush over the mycelium with a sterile inoculation loop. During this process, the spores detached and could be transferred to a sterile vessel via a funnel covered with sterile gauze [28]. The filtrate containing the spores (1 x 10^10^ CFU/ml) was then centrifuged at 9,000 rpm for 10 minutes and washed twice with the same solution to dissolve the spores. For the inactivation experiment, this solution was diluted until approximately 4 x 10^6^ spores were present in 1 mL of 0.9% NaCl solution without TWEEN 80.

**Figure 1.**
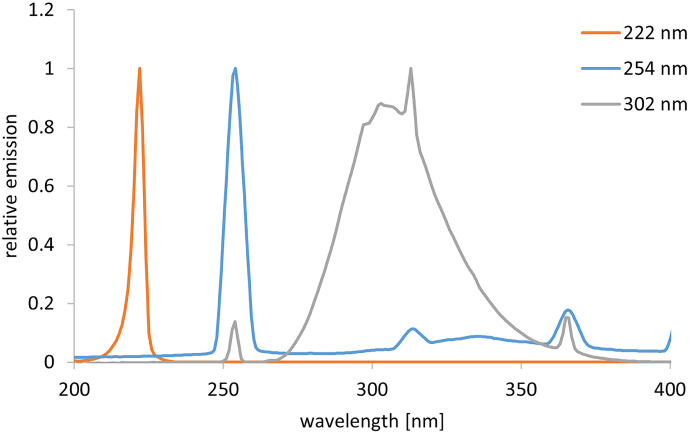
Relative emission spectra of the different radiation sources.

The inactivation experiments were performed with different radiation sources and different wavelengths. A krypton chloride excimer lamp (Ushio Care 222 Modell B1, Ushio Europe B.V.) with an irradiation intensity of 0.25 mW/cm^2^ was employed for irradiation at 222 nm and a low pressure mercury vapor lamp (TUV 15W/G15T8, Philips) with an intensity of 0.23 mW/cm^2^ was applied for irradiation at 254 nm. For irradiation in the UVB range (302 nm), an irradiation unit (UVP 3UV Lamp, Analytik Jena GmbH+Co. KG) with an irradiation intensity of 7.4 mW/cm^2^ was selected. The corresponding spectra can be found in Figure 1. The radiation source was placed over a Petri dish containing 3 mL of the sample. The control sample was placed next to the irradiated sample in the setup, covered with aluminum foil to prevent irradiation. The irradiance was measured with a photometric detector (X1-UV-3727 with X1 measuring device, Gigahertz-Optik GmbH) at the respective wavelengths (222 and 254 nm), and the UVB irradiance was determined with a spectroradiometer (CAS 140D with integrating sphere of Instrument Systems) prior to the experiments. To prevent possible photoreactivation, all samples (control and irradiation samples) and spread plates were covered with aluminum foil except during irradiation.

All experiments were performed at room temperature. After fixed periods of time, 100 µL samples of the fungal suspension were taken and the irradiation was then continued. Three samples were taken at each of the fixed times and 3 independent experimental runs were performed.

After an incubation period of 48 hours, the grown yeast colonies and after 96 hours, the germinated spores of the mold could be counted and the colony forming unit per mL were determined. The resulting logarithmic reduction values were calculated in relation to the initial value and could be graphically displayed with respective linear fit curves. Furthermore, the experiments were repeated at least 3 times with multiple dilution levels plated out and evaluated for each measurement point. The inactivation rate constants (k values) were determined from 1-log reduction doses and the reduction according to the calculation method of Lemons et al [29].

For comparison, 1-log reduction doses of various members of the genus *Candida, S. cerevisiae* and also of *C. cladosporioides, A. niger,* and *A. fumigatus* were selected from other published studies. As selection criterion, an open setup (Petri dishes as vessels) under aerobic condition was taken. Furthermore, the irradiation medium should have been PBS, water, or similar transparent solutions. The 1-log reduction doses were taken directly from text or tables in the literature or read from the graphs. The collected D90 values were then presented as a boxplot using Origin 2021b (Originlab Corporation).

## RESULTS

**Figure 2.**
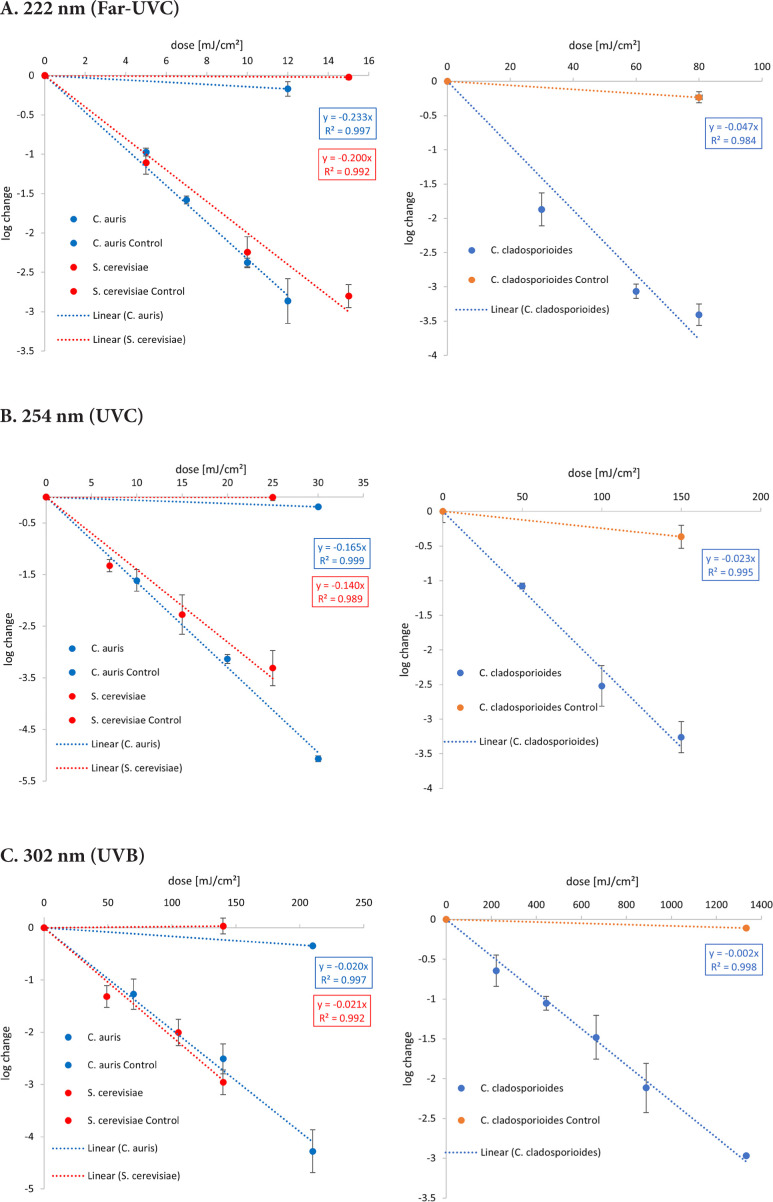
**Overview of the inactivation curves for *C. auris, S. cerevisiae* and *C. cladosporioides*.** The irradiation was carried out with different wavelengths — (A) Far-UVC, (B) UVC, and (C) UVB.

The graphical representation of the irradiation results including the log reduction against the irradiation dose (mJ/cm^2^) and the ensuing linear fit can be found in Figure 2. The determined 1-log reduction doses of each wavelength and microorganism are given in Table 1.

**Table 1.** 1-Log Reduction Doses from Literature and From This Study (highlighted) in Liquid Media with Similar Experimental Conditions

Microorganism	Wavelength (nm)	1-Log reduction dose (mJ/cm^2^)	k values (cm^2^/mJ)
*Candida albicans*	222	median value: 9.55	0.24
		9.2 [30]	0.25
		9.9 [31]	0.23
	254	median value: 11.8	0.20
		11.8 [32]	0.20
		7.6 [31]	0.30
		6.5 [33]	0.35
		16.0 [30]	0.14
		17.0 [30]	0.14
		19.0 [30]	0.12
		22.0 [34]	0.10
		9.7 [29]	0.24
		8.3 [29]	0.28
*Candida auris*	222	**4.3** (this study)	**0.54** (this study)
	254	median value: 17.5	0.13
		**6.1** (this study)	**0.38** (this study)
		13.2 [29]	0.17
		22.1 [29]	0.10
		16.7 [29]	0.14
		17.5 [29]	0.13
		18.1 [29]	0.13
		16.4 [29]	0.14
		16.0 [29]	0.14
		15.3 [29]	0.15
		18.1 [29]	0.13
		18.5 [29]	0.12
		22.7 [35]	0.10
		24.9 [35]	0.09
	302	**51.3** (this study)	**0.04** (this study)
*Candida kofuensis*	254	median value: 9.4	0.24
		10.6 [36]	0.22
		9.4 [36]	0.24
		7.8 [36]	0.30
*Candida parapsilosis*	254	9.8 [37]	0.23
*Candida guilliermondii*	254	50.0 [38]	0.05
	302	5,000 (313 nm) [38]	0.0005
*Candida utilis*	254	80.0 [38]	0.03
	302	4,000 (313 nm) [38]	0.0006
*Saccharomyces cerevisiae*	222	**5.0** (this study)	**0.46** (this study)
	254	median value: 7.1	0.32
		**7.1** (this study)	**0.32** (this study)
		16.7 [39]	0.14
		21.2 [40]	0.11
		5.2 [41]	0.44
		33.0 [42]	0.07
		6.3 [43]	0.37
		2.5 [44]	0.92
	302	median value: 59.9	0.04
		**47.8** (this study)	**0.05** (this study)
		72.0 [21]	0.03
*Cladosporium cladosporioides* spores	222	**21.2** (this study)	**0.11** (this study)
	254	median value: 233.4	
		**44.1** (this study)	**0.05** (this study)
		270.0 [34]	0.009
		233.4 [45]	0.01
	302	median value: 357.9	0.006
		**434.8** (this study)	**0.005** (this study)
		281.0 [45]	0.008
*Aspergillus niger* spores	222	median value: 85.0	0.03
		106.8 [31]	0.02
		72.5 [32]	0.03
		85.0 [46]	0.03
	254	median value: 57.9	0.04
		121.9 [31]	0.02
		123.3 [46]	0.02
		50.8 [32]	0.05
		122.0 [47]	0.02
		65.0 [48]	0.04
		60.0 [49]	0.04
		190.0 [34]	0.01
		42.0 (265 nm) [48]	0.05
		18.0 (280 nm) [48]	0.13
		15.0 (280 nm) [49]	0.15
*Aspergillus fumigatus spores*	254	median value: 16.8	0.14
		3.1 [50]	0.74
		30.4 [51]	0.08

Lower 1-log reduction doses are observed for *C. auris* at 222 nm and 254 nm with 4.3 and 6.1 mJ/cm^2^ than for *S. cerevisiae* with 5.0 and 7.1 mJ/cm^2^, respectively. Irradiating with UVB, a higher irradiation dose for a reduction of 90% is needed for *C. auris* with 51.3 mJ/cm^2^ than for *S. cerevisiae* with 47.8 mJ/cm^2^.

Compared with both yeasts, a higher 1-log reduction dose was needed to inactivate *C. cladosporioides* for each wavelength with 21.2 mJ/cm^2^ at 222 nm, 44.1 mJ/cm^2^ at 254 nm, and 434.8 mJ/cm^2^ for UVB. The difference between the 1-log reduction doses increased with higher irradiation wavelengths.

Table 1 lists literature and this study’s experimental results for 1-log reduction doses of yeasts (various *Candida* species and *S. cerevisiae*) and spores of molds (*C. cladosporioides, A. niger*, and *A. fumigatus*) with respect to photoinactivation by different UV wavelengths (222, 254, and 302 nm).

Most data were found for irradiation at 254 nm. Furthermore, data could be retrieved for different representatives of the genus Candida. Most 1-log reduction doses for photoinactivation were published for *C. albicans* and *C. auris*.

**Figure 3.**
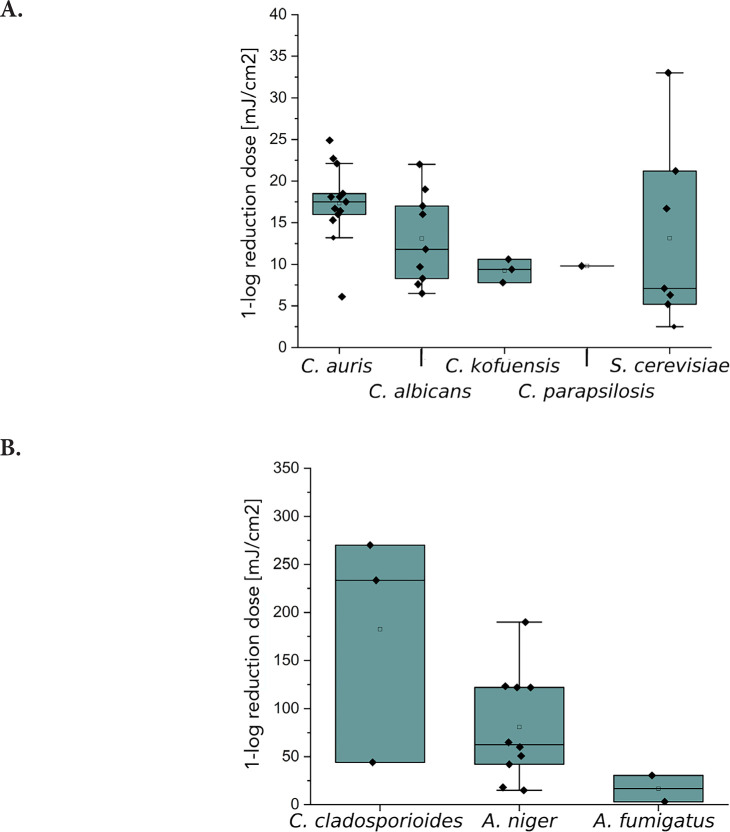
**Representation of 1-log reduction doses with irradiation at 254 nm of different *Candida* species as well as *S. cerevisiae* (A) and spores of molds like *C. cladosporioides, A. niger*, and *A. fumigatus* (B).** The boxplots were created based on data (Table 3) from literature and measurements from this study.

Figure 3 represents literature values and this study’s experimental results as a boxplot for irradiation at 254 nm. For *C. albicans*, the irradiation doses for a D90 reduction are very diverse and range from 6.5-22 mJ/cm^2^ and include reduction doses that are also needed for other Candida species, such as *C. auris, C. davisiana, C. kofuensis*, and *C. parapsilopsis* (Figure 3A). For *C. guilliermondii* and *C. utilis*, high 1-log reduction doses of 50.0 and 80.0 mJ/cm^2^ were determined [38]. For *S. cerevisiae*, 1-log reduction doses were measured and determined from literature sources, which range from 2.5-33.0 mJ/cm^2^.

For molds (Figure 3B), the most 1-log reduction doses were noted for *A. niger*, ranging from 15.0-190.0 mJ/cm^2^. Values from 44.1-270.0 mJ/cm^2^ formed the boxplot for *C. cladosporioides*. The 1-log reduction doses for *A. fumigatus* are 3.1-30.4 mJ/cm^2^ for irradiation at 254 nm.

## DISCUSSION

Comparing the 1-log reduction doses in Table 3, most have been retrieved for irradiation at 254 nm. Comparable studies at other wavelength ranges, such as Far-UVC and UVB, were rare. In general, for all fungi and all irradiation wavelengths, exponential photoinactivation could be observed with rising 1-log reduction doses for higher wavelengths.

For 222 nm, a 1-log reduction dose of 4.3 mJ/cm^2^ was determined for *C. auris* in this study and is one of the first inactivation experiments at this wavelength. This dose is lower than the resulting median from various studies for *C. albicans* with a 1-log reduction dose of 9.55 mJ/cm^2^ at 222 nm in liquids. (Other studies focusing on surface disinfection using Far-UVC, like a recent investigation by Memic et al [52], were not included in the analysis, because of the incomparable experimental conditions.) For *S. cerevisiae* in suspension, a dose 1.2 times higher than for *C. auris* was determined with 5.0 mJ/cm^2^. When irradiated at 302 nm (broad-band UVB), very similar 1-log reduction doses of 51.3 and 47.8 mJ/cm^2^ were observed for *C. auris* and *S. cerevisiae* in liquid media, respectively (Figure 2).

In Table 1, the high D90 reduction doses at 254 nm with 50.0 and 80.0 mJ/cm^2^ of *C. utilis* and *C. guilliermondii* are particularly striking [38]. *C. utilis* is used in food industry and is classified as harmless [53–55]. *C. guilliermondii* has a low pathogenicity and can be found in biotechnology [56–58]. However, the amount of data from *C. utilis* and *C. guilliermondii* is too small to make reliable statements about a possible dependence of the pathogenicity. In comparison to other yeast, such as *S. cerevisiae*, which is also non-pathogenic, but for which similar 1-log reduction doses have been measured (Figure 2 and Figure 3), no dependence of pathogenicity on the irradiation dose can be demonstrated.

Figure 3 presents the literature and experimental 1-log reduction doses by irradiation at 254 nm. In Figure 3A, all literature values of *C. auris* are above the median value of *C. albicans* by a factor of 0.91. *C. kofuensis* and *C. parapsilosis* are below the median value of *C. albicans*. Furthermore, statements can be made about possible surrogates for *C. auris* and *C. albicans*. Since the 75% quantile of *C. albicans* is between the median and the 25% quantile of *C. auris, C. auris* could be described as a possible surrogate for *C. albicans* when irradiated at 254 nm. The box-forming values of *S. cerevisiae* are widely scattered and include all 1-log reduction doses of the other yeasts. However, the median and thus also the 25% quantile of the box is below the 25% quantile of the other yeasts. For this reason, *S. cerevisiae* is not a good surrogate for *C. albicans* for photoinactivation at 254 nm.

Both, Table 1 and Figure 3B reveal that much higher D90 doses are required for spores of the molds *C. cladosporioides, A. niger,* and *A. fumigatus* than for the yeasts. The 1-log reduction dose for *C. cladosporioides* with 434.8 mJ/cm^2^ is 10 times higher than for *C. auris* with 51.3 mJ/cm^2^ when irradiated at 302 nm. In Table 1, it is noticeable that hardly any data on photoinactivation are available for molds at the selected irradiation wavelengths. Especially for *A. fumigatus*, only 2 literature values are published for irradiation by UVC [50, 51]. No literature values are available for *C. cladosporioides* when irradiated by Far-UVC. In this study, a 1-log reduction dose of 21.2 mJ/cm^2^ was determined for this fungus, which is 4 times lower than the median value for *A. niger* with 85.0 mJ/cm^2^. For irradiation with UVB, only data for *C. cladosporioides* exists.

Figure 3B presents 1-log reduction doses from Table 1 as a boxplot for an irradiation at 254 nm. It is notable that the D90 values for all 3 molds differ from each other. The irradiation doses for *A. niger* and *A. fumigatus* are below the mean value and also partly below the measured value for *C. cladosporioides.* The recorded inactivation doses for *C. cladosporioides* are 44.1 mJ/cm^2^ up to 270.0 mJ/cm^2^ [34]. The median 1-log reduction dose for *A. niger* is 57.9 mJ/cm^2^ and the irradiation doses for *A. fumigatus* are 3.1 mJ/cm^2^ and 30.4 mJ/cm^2^ [50, 51]. For *C. cladosporioides* and *A. niger*, the median values for 1-log reduction doses differ by a factor of 2.7 at 254 nm. With regard to possible surrogate properties, this can be ruled out for *A. niger* on the aspect of photoinactivation at 222 nm of *C. cladosporioides*. This is also the case for irradiation at 254 for all 3 molds in relation to each other. For irradiation with UVB and irradiation at 222 nm with regard to *A. fumigatus* and *C. cladosporioides*, it is not possible to make a surrogate statement due to a lack of data.

There are various investigations for possible applications for the 3 wavelength ranges analyzed here. There is a broad spectrum of therapeutic approaches for various diseases (eg, skin diseases), possible disinfection systems and applications in the food industry [52, 59–61]. Even if many of these approaches can cure diseases or prevent infections in advance through disinfection, there are no clear statements to the question of how much the irradiation reduces the number of infections [62].

## CONCLUSION

With regard to possible surrogates, the investigations carried out here described that *S. cerevisiae* represents a possible irradiation surrogate for *C. auris* for all 3 wavelengths. It was also possible to describe by comparing the literature and the resulting boxplot for irradiation at 254 nm that *S. cerevisiae* and *C. kofuensis* could be a surrogate for *C. albicans* for UVC irradiation experiments.

Due to the low number of photoinactivation data to be compared, further investigations should be performed on other fungi, both yeasts and molds. It is also important to carry out irradiation not only with UVC, but also with UVB and especially Far-UVC. Far-UVC has a great potential for application in the presence of people due to its low penetration depth and there are already methods for curing skin diseases with UVB. To obtain further information on possible surrogates or on the possible connection between pathogenicity and radiation sensitivity, more inactivation data must be available for the respective fungi.
